# A T cell–based SARS-CoV-2 spike protein vaccine provides protection without antibodies

**DOI:** 10.1172/jci.insight.155789

**Published:** 2024-01-23

**Authors:** Juan Shi, Jian Zheng, Xiujuan Zhang, Wanbo Tai, Ryan Compas, Jack Deno, Natalie Jachym, Abhishek K. Verma, Gang Wang, Xiaoqing Guan, Abby E. Odle, Yushun Wan, Fang Li, Stanley Perlman, Liang Qiao, Lanying Du

**Affiliations:** 1 Institute for Biomedical Sciences, Georgia State University, Atlanta, Georgia, USA.; 2 Department of Microbiology and Immunology, and Department of Pediatrics, University of Iowa, Iowa City, Iowa, USA.; 3 Lindsley F. Kimball Research Institute, New York Blood Center, New York, New York, USA.; 4 Department of Microbiology and Immunology, Stritch School of Medicine, Loyola University Chicago, Maywood, Illinois, USA.; 5 Department of Pharmacology, University of Minnesota Medical School, Minneapolis, Minnesota, USA.; 6 Center for Coronavirus Research, University of Minnesota, Minneapolis, Minnesota, USA.

**Keywords:** COVID-19, Vaccines, Adaptive immunity, T cells

## Abstract

SARS-CoV-2 spike–based vaccines are used to control the COVID-19 pandemic. However, emerging variants have become resistant to antibody neutralization and further mutations may lead to full resistance. We tested whether T cells alone could provide protection without antibodies. We designed a T cell–based vaccine in which SARS-CoV-2 spike sequences were rearranged and attached to ubiquitin. Immunization of mice with the vaccine induced no specific antibodies, but strong specific T cell responses. We challenged mice with SARS-CoV-2 wild-type strain or an Omicron variant after the immunization and monitored survival or viral titers in the lungs. The mice were significantly protected against death and weight loss caused by the SARS-CoV-2 wild-type strain, and the viral titers in the lungs of mice challenged with the SARS-CoV-2 wild-type strain or the Omicron variant were significantly reduced. Importantly, depletion of CD4^+^ or CD8^+^ T cells led to significant loss of the protection. Our analyses of spike protein sequences of the variants indicated that fewer than one-third presented by dominant HLA alleles were mutated and that most of the mutated epitopes were in the subunit 1 region. As the subunit 2 region is conservative, the vaccines targeting spike protein are expected to protect against future variants due to the T cell responses.

## Abstract 2

A vaccine that iduces only SARS-CoV-2-S-specific T cells provides marked protection against viral challenge, supporting effectiveness of current COVID-19 vaccines against SARS-CoV-2 variants.

## Graphical abstract

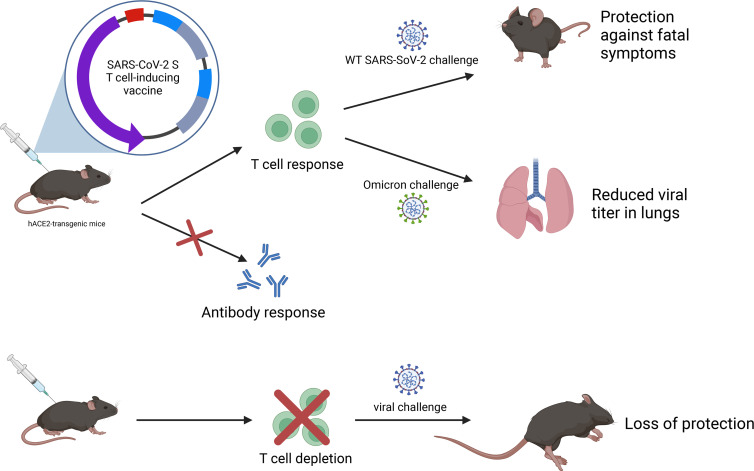

## Introduction

Coronavirus disease 2019 (COVID-19) emerged in December 2019. The virus that causes COVID-19, severe acute respiratory syndrome coronavirus-2 (SARS-CoV-2), was sequenced and published in January, 2020 (1). SARS-CoV-2 caused a pandemic, with consequences much more severe than its close relative SARS-CoV-1, which was identified in 2002 (2). Global collaborative efforts have been made by pharmaceutical companies, academic laboratories, and governmental agencies, resulting in rapid development of vaccines to prevent SARS-CoV-2 infection or symptoms. Many of the vaccines are aimed at inducing immune responses (mostly neutralizing antibodies) to the spike (S) protein of SARS-CoV-2, and the S protein sequence in the initial vaccines was from the original virus strain (3–10). Some of the vaccines have been approved in a number of countries, which have been successful in reducing COVID-19 (9–12). However, SARS-CoV-2 variants of concern have been reported, e.g., Alpha (B.1.1.7), Beta (B.1.351), Gamma (P.l), Delta (B.1.617.2), and Omicron (BA.1.1.529 and its subvariants) (13–17). It is expected that more variant strains will appear as the virus keeps mutating when replicating. The variant strains have deletions or substitutions in the S protein.

The S protein has 2 subunits, S1 and S2, which are responsible for receptor angiotensin-converting enzyme 2 (ACE2) binding and membrane fusion, respectively (2, 18). As the neutralizing antibodies are mostly against the receptor-binding domain (RBD) of the S1 subunit, the mutations in the RBD lead to a reduction in neutralizing activity of immune sera from individuals vaccinated with SARS-CoV-2 vaccines or from convalescent COVID-19 patients (19–22). This raises the burning question whether the current vaccines will still protect against emerging variant strains without significant amounts of neutralizing antibodies or whether we should provide boost immunizations using mutated S proteins such as the current bivalent vaccines from Moderna and Pfizer-BioNTech.

Although it is believed that neutralizing antibodies are the primary immune effector to provide viral protection, cellular immunity (CD4^+^ T cells and CD8^+^ T cells) also plays important roles in controlling viruses. We have shown that cytotoxic T lymphocytes (CTLs) alone are sufficient to provide full protection against Zika virus–induced fetal damage (23). Thus, we addressed the question of whether the current vaccines targeting the S protein will still provide protection in the absence of an optimal SARS-CoV-2 antibody response. To this end, we designed a T cell–based S protein vaccine that induces only T cell immunity but no antibodies, and tested whether it protects against SARS-CoV-2 challenge. Generation of virus-specific CD8^+^ T cells depends on the presentation of epitopes (~9 amino acids) in the context of MHC class I. Usually, intracellular proteins subject to continuous turnover are degraded at different rates into short peptides in the proteasome. Peptides generated in the proteasome are transported by peptide transporters to the endoplasmic reticulum for loading onto MHC class I. The intracellular proteins targeted for proteolysis often have ubiquitin attached to them. Ubiquitin-protein conjugates are degraded by the proteasome. It has been shown that ubiquitination of a viral protein greatly enhanced degradation of the viral protein and consequently caused an enhanced induction of specific CTLs (23, 24). Accordingly, we made the T cell–based SARS-CoV-2 vaccine by rearranging the S gene to disrupt the S protein conformation and adding a ubiquitin gene to enhance S protein degradation in the proteasome for effective CTL generation. We showed that the vaccine induced only specific T cell responses without specific antibodies and provided protection in mouse models.

## Results

### Analysis of T cell epitope mutations in S protein.

We analyzed the conservation of human T cell epitopes in S proteins, including MHC class I– and class II–restricted epitopes in the variants of concern. Initially, we analyzed the predicted epitopes presented by the most prevalent MHC class I and class II alleles. The top 6 most frequently occurring alleles were selected (Supplemental Tables 1 and 2; supplemental material available online with this article; https://doi.org/10.1172/jci.insight.155789DS1). TepiTool, a T cell epitope prediction resource from the Immune Epitope Database (IEDB), was used to find the top 10 highest scoring epitopes of the S protein from the original strain (GenBank accession number QHR63250.2) for each allele based on the 50% inhibitory concentration (IC_50_) value, and then we determined the number of mutated epitopes in the variants of concern. We found that the T cell epitopes presented by MHC class I alleles were more conserved than those presented by MHC class II alleles in these variants; on average, 3.7% of epitopes presented by HLA-A alleles, 6.7% of those by HLA-B alleles, 5.7% of those by HLA-C alleles, 15.7% of those by HLA-DR alleles, 9.7% of those by HLA-DQ alleles, and 24% of those by HLA-DP alleles were mutated (Supplemental Figure 1, A–F, and Supplemental Figure 2, A–F). Then, we analyzed a list of confirmed T cell epitopes presented by all MHC alleles based on published studies (25–31) and found a similar trend; the MHC class I epitope mutation rate was approximately 6.4% and the MHC class II epitope mutation rate was approximately 11.5% (Supplemental Figure 1, G and H, and Supplemental Figure 2, G and H). Most of the mutations are located in the epitopes in the S1 subunit. Thus, the data suggest that T cell responses to S protein may survive the mutations.

### Construction of a T cell–based SARS-CoV-2 Ub-S DNA vaccine.

**Figure 1:**
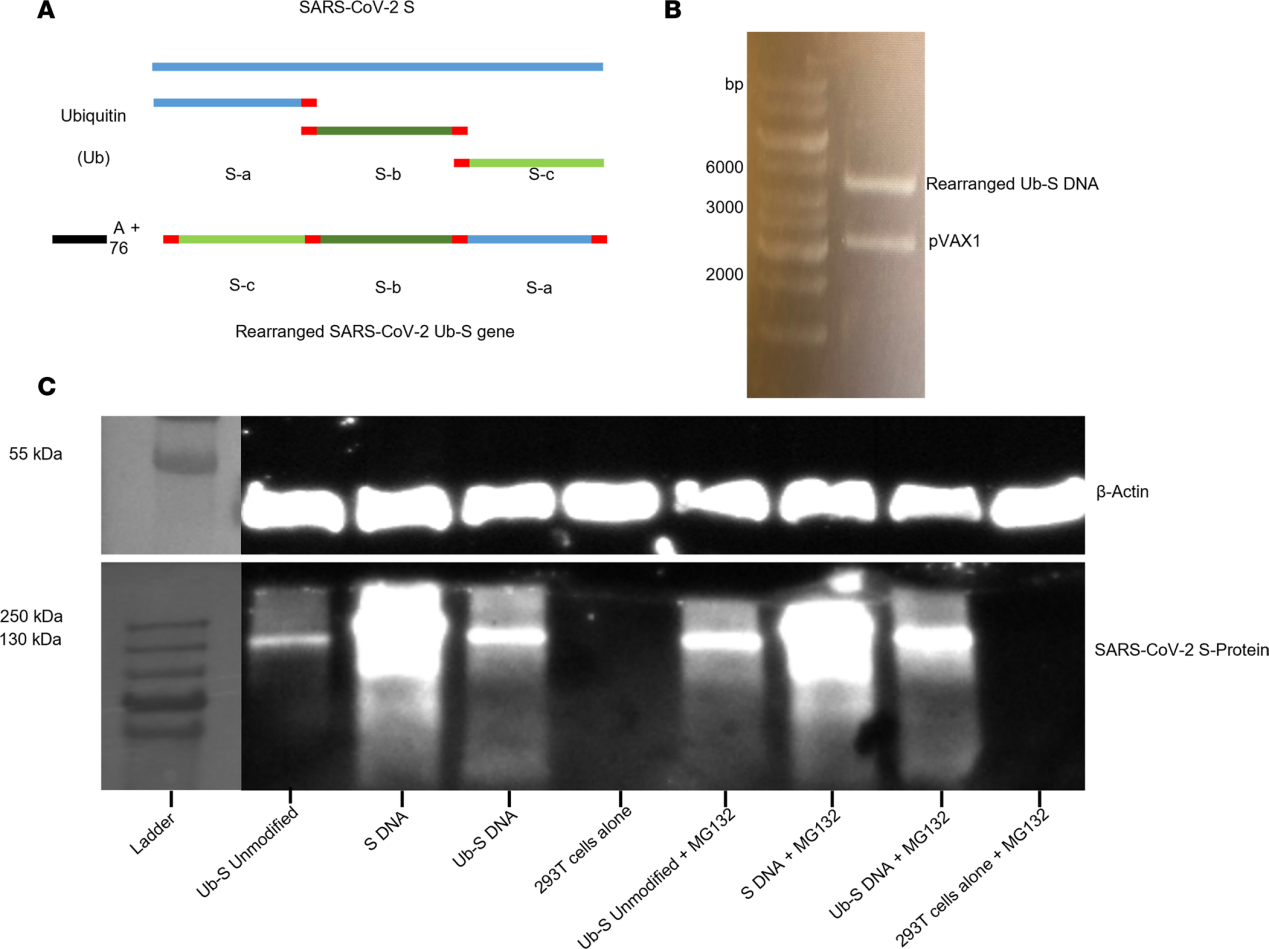
SARS-CoV-2 T cell–based Ub-S DNA vaccine design and antigen expression. (**A**) Schematic diagram of plasmid design. The gene sequence encoding the S protein was split into 3 parts (denoted S-a, S-b, and S-c). The 30 nucleotide bases before and after any cleaved site were placed back to preserve any epitopes that may have been disrupted. The open-reading frame gene encoding a human monomer of ubiquitin (Ub) was placed immediately upstream of the rearranged S sequence. The gene encoding a glycine at the 76th residue was modified to encode an alanine to enhance the stability of the Ub-S complex. (**B**) The plasmids were digested with EcoRI and NotI and run in an agarose gel. (**C**) 293T cells were transfected with the Ub-S plasmid encoding ubiquitinated and rearranged S protein, a Ub-S Unmodified plasmid encoding ubiquitinated original S protein, or a plasmid encoding original S protein (no ubiquitin), overnight. The cells were allowed to stably express plasmid for 36 hours. After this period, MG132 was added overnight (right). The cell lysate was analyzed via Western blotting for the expression of SARS-CoV-2 S protein and β-actin as a control. Lanes were run on the same respective gels, but are noncontiguous.

Because gene rearrangement usually changes the original folding of a protein and prevents induction of antibodies, in particular those against conformational epitopes, we split the SARS-CoV-2 S gene into 3 parts (segments a, b, and c) and rearranged them (segment c/b/a ratio is 1539:1425:1290) (Figure 1A). Thirty nucleotides before and after cleaved sites were added back in order to preserve CTL epitopes that may have been disrupted. To enhance degradation of the rearranged S protein, we added a ubiquitin gene to the construct. The ubiquitin gene can be mutated to encode ubiquitin with an alanine at residue 76 to enhance ubiquitin-protein complex stability (24). Because human and mouse ubiquitin genes share the same sequences, we fused the gene encoding a monomer of human mutated ubiquitin to the 5′ end of the rearranged *S* DNA sequence, with its glycine (G) at the 76th residue replaced by alanine (A) to enhance stability of the Ub-S complex (Ub-S DNA) (Figure 1A). The rearranged SARS-CoV-2 Ub-S DNA sequence was inserted into the pVAX1 vector and digested to confirm the correct size (Figure 1B). We also made 2 additional constructs expressing either the full-length original S protein without ubiquitin (S DNA) or the full-length original S protein fused to ubiquitin (Ub-S Unmodified) as the controls for rearranged Ub-S DNA. We transfected 293T cells with the plasmids and cultured the cells in the presence and absence of the proteasome inhibitor MG312 and performed Western blotting analyses. As shown in Figure 1C, the ubiquitinated and rearranged S protein (Ub-S DNA) and ubiquitinated original S protein (Ub-S Unmodified) were reduced as compared with the original S protein without ubiquitin. The expression of these 2 ubiquitinated proteins was enhanced in the presence of MG 312, suggesting that ubiquitinated proteins are degraded in the proteasome. There was no significant difference at the protein level between Ub-S DNA and Ub-S Unmodified, indicating that gene arrangement has no impact on the protein degradation.

### SARS-CoV-2 Ub-S DNA vaccine induced strong specific T cell responses without antibodies.

In the next sets of experiments, we tested the short-term and long-term immune responses induced by the Ub-S DNA vaccine expressing the ubiquitinated and rearranged S protein in different mouse strains and various adjuvants.

**Figure 2:**
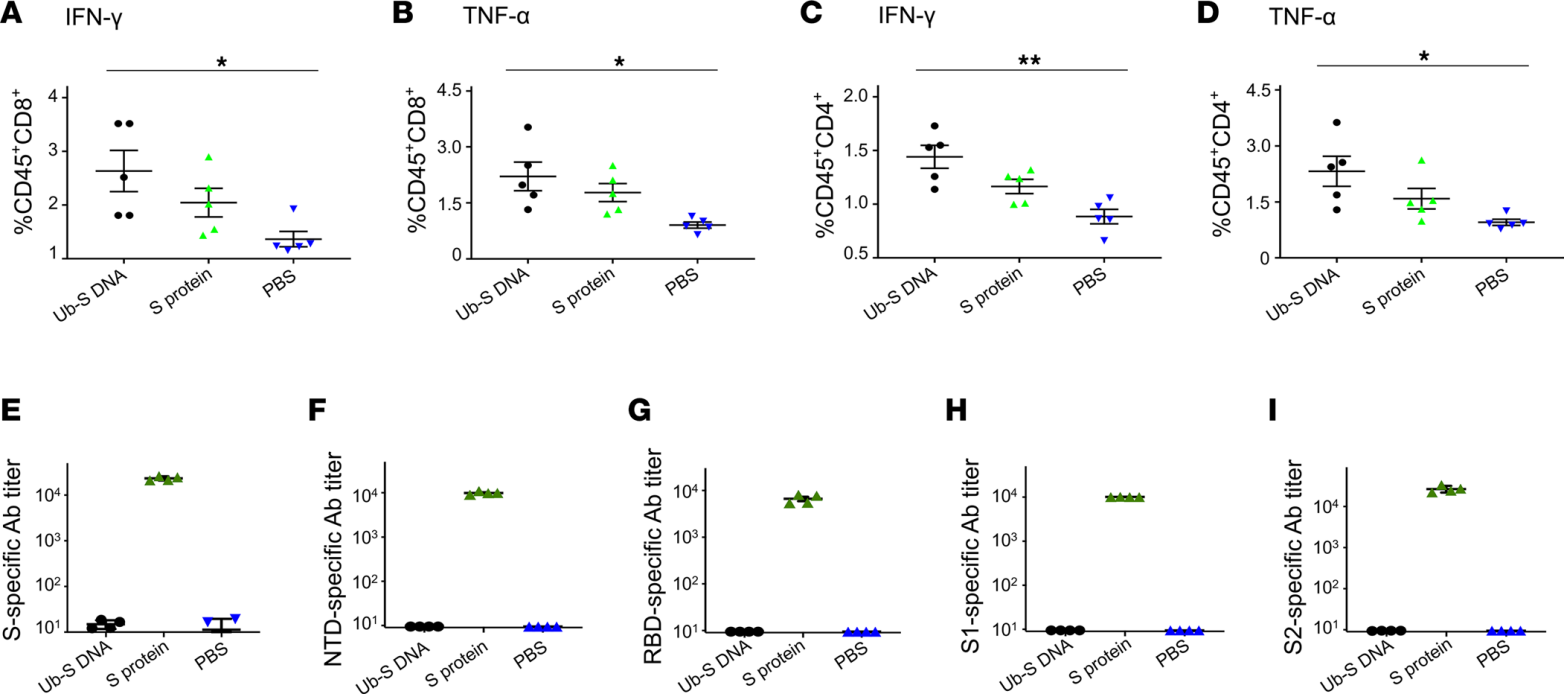
SARS-CoV-2 Ub-S DNA vaccine induced strong T cell responses without antibodies in BALB/c mice. Mice were immunized with Ub-S DNA vaccine (plus imiquimod adjuvant), full-length S protein vaccine control (plus Alum + MPL adjuvants), or PBS without adjuvant (background control), and boosted twice with the same immunogens plus the respective adjuvants at 3-week intervals. Ten days after the third dose, splenocytes from the mice were tested for S-specific T cell responses, and sera were tested for IgG antibodies specific for the S, N-terminal domain (NTD), receptor-binding domain (RBD), S1, or S2 of SARS-CoV-2. SARS-CoV-2 S–specific CD8^+^ (**A** and **B**) and CD4^+^ (**C** and **D**) T cells were analyzed by flow cytometry. Splenocytes were stimulated with pooled peptides (final concentration 5 μg/mL) from the SARS-CoV-2 S protein predicted to contain both CTL and T helper cell epitopes, and IFN-γ– and TNF-α–producing CD45^+^CD8^+^ T cells and IFN-γ– and TNF-α–producing CD45^+^CD4^+^ T cells were stained for respective cell surface and intracellular cytokine markers. (**E**–**I**) Serum SARS-CoV-2 S–, NTD–, RBD–, S1–, or S2–specific IgG antibodies were tested by ELISA. The ELISA plates were respectively coated with SARS-CoV-2 full-length S protein or its fragments (1 μg/mL), and antibody (Ab) titers were calculated as the endpoint dilution that remained positively detectable. Data are expressed as mean ± SEM of individual samples (for T cell detection) or quadruplicate wells from pooled sera of mice in each group (*n* = 5). *P < 0.05, **P < 0.01 by ordinary 1-way ANOVA with Tukey’s multiple-comparison test. Experiments were repeated twice, and similar results were obtained.

First, young adult BALB/c mice (6–8 weeks old) were immunized with this DNA vaccine in the presence of the TLR7 agonist imiquimod adjuvant, or SARS-CoV-2 full-length S protein (S protein) in the presence of aluminum (Alum) plus monophosphoryl lipid A (MPL) adjuvants, and boosted twice at a 3-week interval. Here, different adjuvants were used, as they have been optimized for DNA and proteins, respectively (23, 32). Mice were sacrificed 10 days after the third immunization, and spleens and sera were collected to determine the T cell responses to SARS-CoV-2 S protein and antibody responses to the S protein or different S protein fragments. The results indicated that full-length S protein induced both CD8^+^ and CD4^+^ T cells that produced IFN-γ and TNF-α in response to stimulation with S peptides (Figure 2, A–D, and Supplemental Table 3) and IgG antibodies specific to SARS-CoV-2 S protein and its fragments, including the N-terminal domain (NTD), receptor-binding domain (RBD), S1, and S2 (Figure 2, E–I). In contrast, the Ub-S DNA vaccine expressing the rearranged and ubiquitinated S protein induced similar levels of CD8^+^ and CD4^+^ T cell responses to those of the full-length S protein control vaccine, but did not induce S-, NTD-, RBD-, S1-, or S2-specific antibodies, unlike the full-length S protein control vaccine (Figure 2, A–I). PBS control only elicited no or background levels of antibody and T cell responses (Figure 2). These data indicate that this DNA vaccine strategy induced CTLs but also T helper cells, probably due to leaking of the rearranged protein into the MHC class II processing pathway. Figure 1 shows that transfection of 293T cells with the plasmid expressing the rearranged and ubiquitinated S protein was present as a thin band without proteasome inhibitor MG312, suggesting that the protein was not fully degraded in the proteasome and then leaked into the MHC class II processing pathway. Because no antibody responses were induced in BALB/c mice, we speculate that no rearranged and ubiquitinated S protein was secreted.

**Figure 3:**
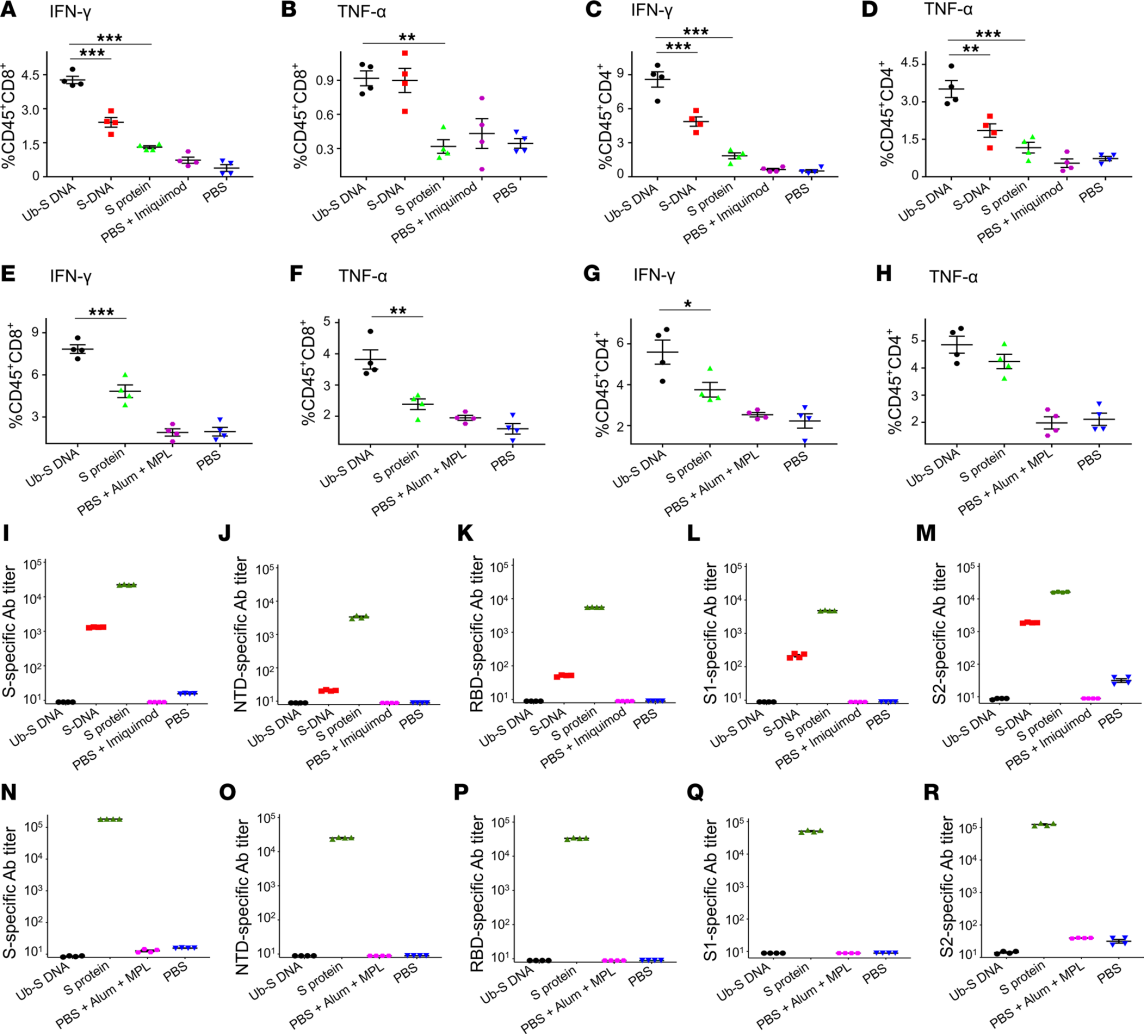
SARS-CoV-2 Ub-S DNA vaccine formulated with different adjuvants induced strong and durable T cell responses without antibodies in C57BL/6 (B6) mice. Mice were immunized with Ub-S DNA vaccine, unmodified S DNA (S DNA) control, full-length S protein (S Protein) vaccine control, or PBS control in the presence of either imiquimod adjuvant or Alum plus MPL adjuvants, and boosted with the same immunogens plus the respective adjuvants twice at 3-week intervals and once at 6 months. PBS without adjuvant was included as a background control. Splenocytes collected 10 days after the last dose from mice immunized with each immunogen plus imiquimod adjuvant (**A**–**D**) or Alum plus MPL adjuvants (**E**–**H**) were analyzed for SARS-CoV-2 S–specific CD8^+^ (**A** and **B** or **E** and **F**) and CD4^+^ (**C** and **D** or **G** and **H**) T cells by flow cytometry. Splenocytes were stimulated with pooled peptides (final concentration 5 μg/mL) from SARS-CoV-2 S protein predicted to contain B6 mouse CTL and Th cell epitopes, and IFN-γ– or TNF-α–producing CD45^+^CD8^+^ and CD45^+^CD4^+^ T cells were stained for respective cell surface and intracellular cytokine markers. Sera collected 10 days after the third dose from mice immunized with each immunogen plus imiquimod adjuvant (**I**–**M**) or Alum plus MPL adjuvants (**N**–**R**) were detected for SARS-CoV-2 S–, NTD–, RBD–, S1–, or S2–specific IgG antibodies by ELISA. The ELISA plates were respectively coated with SARS-CoV-2 full-length S protein, as well as NTD, RBD, S1, or S2 fragment (1 μg/mL), and IgG antibody (Ab) titers were calculated as the endpoint dilution that remained positively detectable. Data are expressed as mean ± SEM of individual samples (for T cell detection) or quadruplicate wells from pooled sera of mice in each group (*n* = 4–5). *P < 0.05, **P < 0.01, ***P < 0.001 by ordinary 1-way ANOVA with Tukey’s multiple-comparison test. Experiments were repeated twice, and similar results were obtained.

Second, to verify these data in a different mouse strain, to identify the immunogenicity of Ub-S DNA in the presence of other adjuvants, and to investigate the booster effect after a longer time, young adult C57BL/6 (B6) mice (6–8 weeks old) were immunized with the DNA vaccine expressing ubiquitinated and rearranged S protein (Ub-S DNA) or full-length S protein (S protein) control vaccine in the presence of imiquimod adjuvant and Alum plus MPL adjuvants, respectively, boosted twice at a 3-week interval and once at 6 months, and then tested for specific antibody responses and T cell responses 10 days after the last immunization. In addition, a DNA containing the original SARS-CoV-2 S sequence without ubiquitin (S DNA) plus imiquimod adjuvant was included as a control for Ub-S DNA. Mice immunized with PBS with or without respective adjuvants were included as background controls. As shown in Figure 3, A–H, full-length S protein adjuvanted with either imiquimod or Alum plus MPL elicited IFN-γ– and TNF-α–secreting CD8^+^ (Figure 3, A, B, E, and F) and CD4^+^ (Figure 3, C, D, G, and H) T cells, in response to stimulation with S peptides for B6 mice (Supplemental Table 4), particularly high-titer IgG antibodies specific for the SARS-CoV-2 S protein, NTD, RBD, S1, and S2 fragments, respectively (Figure 3, I–R). As expected, imiquimod- or Alum plus MPL–adjuvanted Ub-S DNA vaccine expressing the rearranged and ubiquitinated S protein elicited significantly higher and durable CD8^+^ and CD4^+^ T cell responses than the full-length S protein (Figure 3, A–H), but did not elicit any S-, RBD-, NTD-, S1-, or S2-specific antibody responses (Figure 3, I–R). Unlike Ub-S DNA, S DNA expressing the original SARS-CoV-2 S protein without ubiquitin induced both S-specific T cell and antibody responses (Figure 3, A–D and I–M), but the T cell responses were significantly lower, or lower than those induced by Ub-S DNA (Figure 3, A–D). Interestingly, S DNA induced antibody responses only in the presence of imiquimod adjuvant, and the antibodies were undetectable in the presence of Alum plus MPL adjuvants (Figure 3, I–R). In contrast, PBS control with or without adjuvants elicited no or background levels of antibody and T cell responses (Figure 3), and only background levels of T cell responses were elicited in vaccinated and control mice in response to stimulation with unrelated peptides of nonstructural protein 3 (NS3) of Zika virus (ZIKV) (data not shown) (23). These data confirm the ability of Ub-S DNA conjugated with different adjuvants to elicit strong and specific T cell immune responses without antibodies in B6 mice.

### SARS-CoV-2 Ub-S DNA vaccine induced protection against SARS-CoV-2 infection.

**Figure 4:**
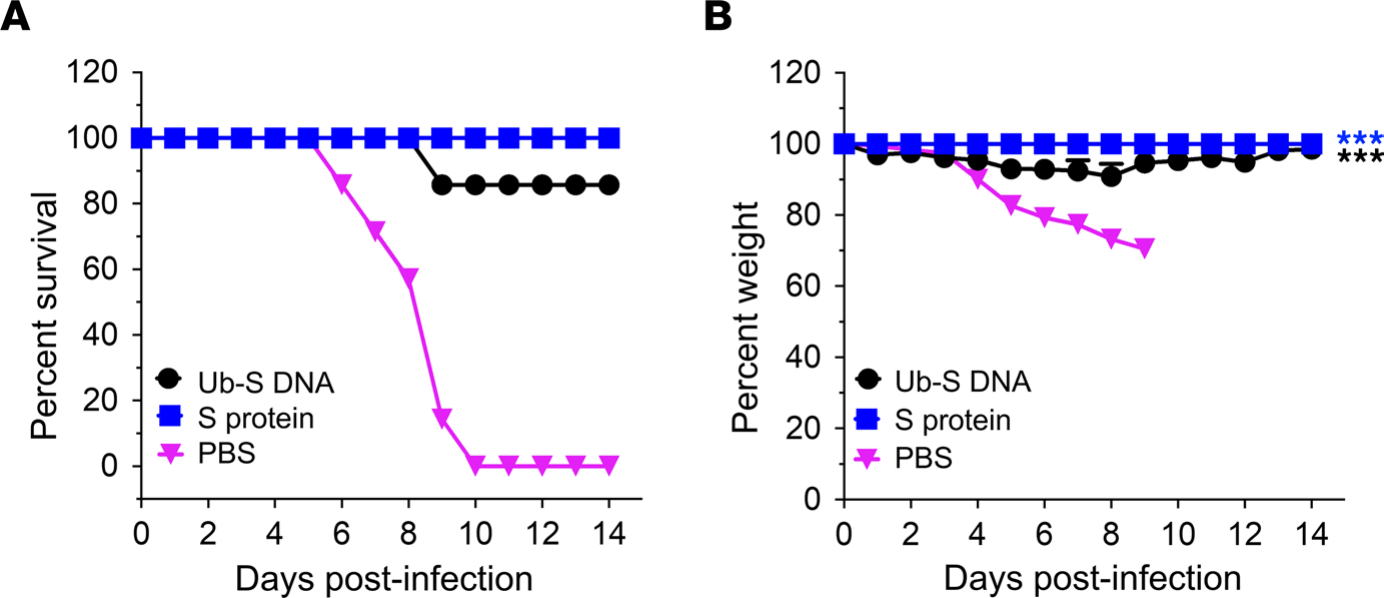
SARS-CoV-2 Ub-S DNA vaccine induced protection against high-dose SARS-CoV-2 infection in mice. hACE2-Tg mice (4–6 months old) were immunized with Ub-S DNA vaccine (plus imiquimod adjuvant), full-length S protein (S Protein) vaccine control (plus Alum + MPL adjuvants), or PBS without adjuvant (background control). Two weeks after the third immunization, mice were i.n. challenged with SARS-CoV-2 (strain 2019n-CoV/USA-WA1/2020, 5,000 PFU/mouse), and recorded for survival (**A**) and weight changes (**B**) for 14 days post infection (p.i.). The data in **B** are presented as mean + SEM of mice in each group (*n* = 7). There was a significant difference in the weight loss after viral challenge between S protein and PBS or Ub-S DNA and PBS groups: ***P < 0.001. Statistical significance of weight loss differences was evaluated starting day 1 after viral challenge using ordinary 1-way ANOVA with Dunnett’s multiple-comparison test. Experiments were repeated once, and similar results were obtained.

To evaluate the protection of SARS-CoV-2 Ub-S DNA vaccine in mature adult mice, we first immunized 4- to 6-month-old mice expressing the SARS-CoV-2 receptor human ACE2 (hACE2-Tg) with this vaccine in the presence of imiquimod adjuvant. The mice were also immunized separately with the SARS-CoV-2 full-length S protein with Alum plus MPL adjuvants, or PBS. Here, different adjuvants were used based on our previously optimized protocols (23, 32). The mice were boosted twice at 3-week intervals. The immunized mice were challenged with a prototypic wild-type SARS-CoV-2 (2019n-CoV/USA-WA1/2020) at a high lethal dose (5,000 PFU/mouse) 2 weeks after booster, and mouse survival and weight loss were monitored for 14 days post infection (p.i.). All mice immunized with full-length S protein survived without weight loss during the monitoring period, whereas 86% of the mice immunized with SARS-CoV-2 Ub-S DNA vaccine survived with minimal weight loss (Figure 4, A and B). In contrast, 100% of the mice in the PBS control group died 10 days p.i. with severe and significant weight loss (Figure 4, A and B). These data demonstrate that, similar to the full-length S protein, the Ub-S DNA vaccine was able to protect mature adult transgenic mice expressing SARS-CoV-2 receptor hACE2 from high-dose SARS-CoV-2 infection.

We then evaluated the protection of SARS-CoV-2 Ub-S DNA vaccine in middle-aged mice. As such, wild-type B6 mice at 8–9 months old were immunized with the Ub-S DNA, full-length S protein, and PBS as described above, challenged with a mouse-adapted SARS-CoV-2 (N501YMA_30_, 5,000 PFU/mouse; not lethal for the mice) 2 weeks after the last boost, and examined for viral titers 2 days p.i. Notably, the Ub-S DNA significantly reduced SARS-CoV-2 titers in the lung compared with the PBS control, although it was not as effective as the full-length S protein vaccine (Supplemental Figure 3A). This is expected, as the Ub-S DNA vaccine did not induce specific antibody responses (as shown below); it can reduce, but not provide, sterilizing immunity. Different from the full-length S protein, which induced high-titer, specific IgG antibodies, Ub-S DNA only elicited a background-level antibody response similar to that induced by PBS control in these mice (Supplemental Figure 3B). The above data indicate that the SARS-CoV-2 Ub-S DNA vaccine significantly reduced viral infection in the challenged middle-aged mice, and that no vaccine-induced antibodies played a role in this protection.

**Figure 5:**
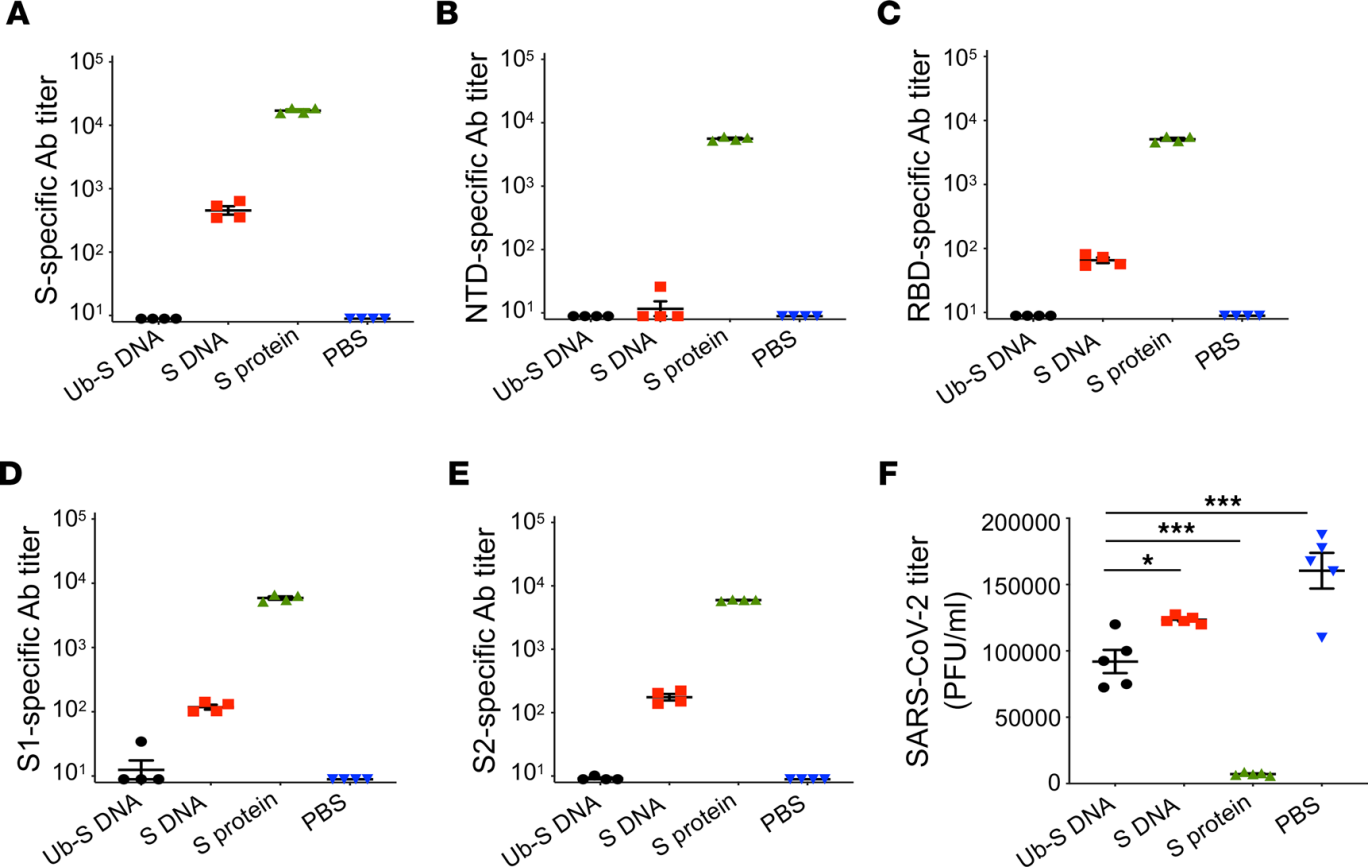
SARS-CoV-2 Ub-S DNA vaccine induced cross-protection against SARS-CoV-2 Omicron BA.5 infection without antibodies in mice. Ten-week-old BALB/c mice were immunized with imiquimod-adjuvanted Ub-S DNA vaccine, unmodified S DNA (S DNA) control, full-length S protein (S protein) control, or PBS control, and boosted twice with the same immunogens at 3-week intervals. Sera were collected from the mice before challenge, and then the mice were i.n. challenged with SARS-CoV-2 (Omicron BA.5 variant, 50,000 PFU/mouse). (**A**–**E**) Sere were analyzed for SARS-CoV-2 S–, NTD–, RBD–, S1–, or S2–specific IgG antibodies by ELISA. The ELISA plates were respectively coated with SARS-CoV-2 full-length S protein, NTD, RBD, S1, or S2 fragment (1 μg/mL), and IgG antibody (Ab) titers were calculated as the endpoint dilution that remained positively detectable. (**F**) Viral titers in the lungs of challenged mice 2 days post infection (p.i.). Data are presented as mean ± SEM of quadruplicate wells from pooled sera or individual samples (for viral titer detection) of mice in each group (*n* = 5). *P < 0.05, ***P < 0.001 indicate significant differences between Ub-S DNA and other groups by ordinary 1-way ANOVA with Dunnett’s multiple-comparison test. Experiments were repeated once, and similar results were obtained.

We further evaluated the cross-protective efficacy of the SARS-CoV-2 Ub-S DNA vaccine against a recent SARS-CoV-2 Omicron variant and compared its protection with that provided by the DNA-containing original S sequence (S DNA) and the original S protein (S protein) in the presence of the same adjuvant. Specifically, young adult BALB/c mice (10 weeks old) were immunized with Ub-S DNA (with ubiquitin), S DNA (without ubiquitin), or full-length S protein, all of which were mixed with imiquimod adjuvant, and challenged with the SARS-CoV-2 Omicron BA.5 variant (50,000 PFU/mouse) 4 weeks after booster. Here, the Omicron BA.5 variant was used because it was a prevalent variant at the time of experiments. BALB/c mice were utilized due to their susceptibility to the Omicron variant, and use of a different viral titer and time point was based on our optimal protocols for Omicron challenge. Sera were collected before challenge for evaluation of IgG antibody responses, and the challenged mice were observed for viral titers in the lungs 2 days p.i. The full-length S protein induced high-titer IgG antibodies, and protected mice against Omicron BA.5 challenge, with significantly reduced viral titers compared with other groups (Figure 5, A–F). The Ub-S DNA also provided effective protection despite a lower efficacy than the full-length protein vaccine (Figure 5F), which is expected because the Ub-S DNA did not induce antibodies (Figure 5, A–E). Nevertheless, the wild-type S DNA induced low-titer IgG antibodies and provided less protection than the Ub-S DNA, resulting in significantly higher viral titers in the lungs of immunized mice than those in the lungs of mice immunized with Ub-S DNA (Figure 5, A–F). These data suggest that the T cell–based Ub-S DNA vaccine induced effective cross-protection against the recent SARS-CoV-2 Omicron variant, and that this protection did not depend on the antibody responses, which is different from that induced with the S protein.

### SARS-CoV-2 Ub-S DNA vaccine–induced T cells played a critical role in protection.

**Figure 6:**
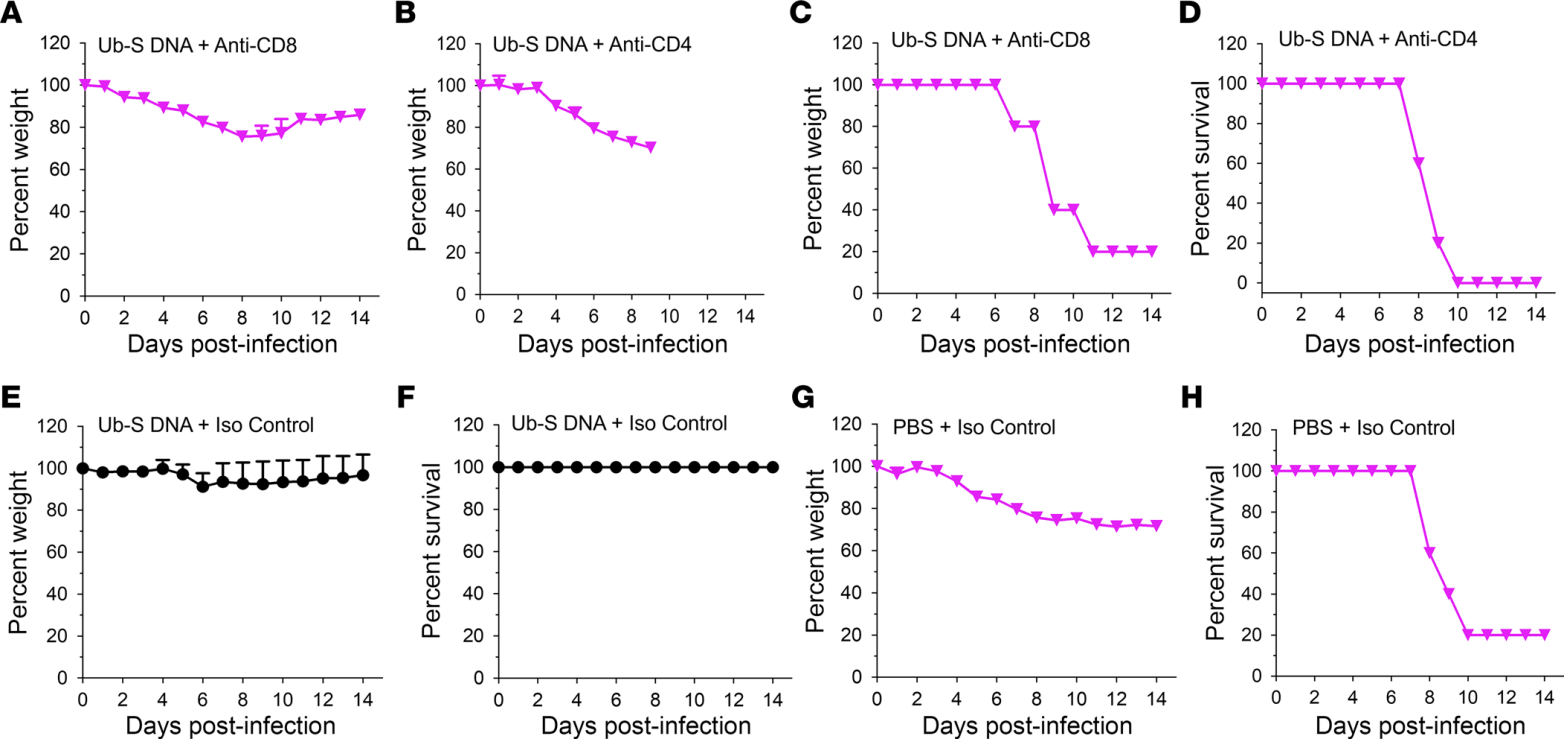
SARS-CoV-2 DNA vaccine–induced T cells provided protection against SARS-CoV-2 infection. hACE2-Tg mice were immunized with imiquimod-adjuvanted Ub-S DNA vaccine or PBS control. Two months after the third immunization, mice were intraperitoneally injected with anti–mouse CD4 (IgG2b, for depleting CD4^+^ T cells), anti–mouse CD8a (IgG2b, for depleting CD8^+^ T cells), or IgG2b isotype antibody control (Iso Ctrl, without depleting CD4^+^ and depleting CD8^+^ T cells) (200 μg/mouse) on days –2, –1, and 1. CD8^+^ or CD4^+^ T cell–depleted mice were i.n. challenged with SARS-CoV-2 (strain 2019n-CoV/USA-WA1/2020, 2,000 PFU/mouse), and recorded for weight changes (**A**, **B**, **E**, and **G**) and survival (**C**, **D**, **F**, and **H**) for 14 days post infection (p.i.) (*n* = 3–5: one group had 3 mice, as 2 mice accidently died before virus infection; all other groups had 5 mice/group). The data in **A**, **B**, **E**, and **G** represent weight changes after viral challenge; mean + SEM of mice in each group was calculated when applicable. There were significant differences in weight loss after viral challenge between the Ub-S DNA–immunized mice receiving isotype antibody control (**E**) and mice receiving anti-CD8 depletion antibody (**A**) (*P* < 0.01), or mice receiving anti-CD4 depletion antibody (**B**) (*P* < 0.05) or PBS-injected mice receiving isotype antibody control (**G**) (P < 0.001). Statistical significance of weight loss differences was evaluated starting day 1 after viral challenge using ordinary 1-way ANOVA with Dunnett’s multiple-comparison test. Experiments were repeated once, and similar results were obtained.

Since SARS-CoV-2 Ub-S DNA did not induce antibodies for preventing SARS-CoV-2 infection, we next determined the contribution of CD8^+^ and CD4^+^ T cells induced by this vaccine to protection against SARS-CoV-2. To this end, mature adult hACE2-Tg mice (4–6 months old) were immunized and challenged with a lethal dose of SARS-CoV-2 (2019n-CoV/USA-WA1/2020, 2,000 PFU/mouse) with or without depletion of CD8^+^ or CD4^+^ T cells 8 weeks after booster, and mouse survival and weight loss were monitored for 14 days p.i. as described above. The depletion of CD4^+^ and CD8^+^ T cells followed our previous protocol, which showed complete depletion of the T cells before challenge in mice (23). The reason for using 2,000 PFU/mouse, instead of a high lethal dose (5,000 PFU/mouse), was to evaluate whether Ub-S DNA–induced T cells can provide complete protection against SARS-CoV-2 infection at a lower lethal dose. Also, the reason for challenging the immunized mice at 8 weeks after the last immunization was to evaluate whether the induced T cells may provide a longer protection against SARS-CoV-2 infection. Indeed, depletion of CD8^+^ or CD4^+^ T cells reduced protection against SARS-CoV-2, with significant weight loss and continuously decreased weight (Figure 6, A and B); 80% and 100% of mice died after CD8^+^ and CD4^+^ T cell depletion, respectively (Figure 6, C and D). In contrast, all mice immunized with the SARS-CoV-2 Ub-S DNA vaccine and treated with isotype antibody control survived without weight loss at the lethal dose tested (Figure 6, E and F). As expected, mice receiving PBS and treated with isotype antibody control had constant weight loss, leading to death, after challenge (Figure 6, G and H). These data demonstrate that SARS-CoV-2 Ub-S DNA vaccine–induced T cells substantially and durably prevent SARS-CoV-2–induced clinical disease, particularly death, and that both CD4^+^ and CD8^+^ T cells play a role in this protection, since depletion of either CD4^+^ or CD8^+^ T cells led to the complete, or almost complete, loss of protection.

## Discussion

SARS-CoV-2 continues to infect humans with high human-to-human transmissibility, and it keeps mutating to generate new variants, calling for the consistent effort to design and develop effective vaccines to prevent infection of the variants of concern and diseases. SARS-CoV-2 S protein–induced neutralizing antibodies are considered key effectors in protecting against SARS-CoV-2 infection (33–39). The role of T cell–induced protection has been demonstrated in animals (40–42). Importantly, patients with X-linked agammaglobulinemia without B cells can recover from SARS-CoV-2 infection (43), providing evidence that T cell immunity alone may provide protection against SARS-CoV-2 in humans. However, the patients infected with SARS-CoV-2 may develop broader T cell responses than the vaccines targeting only S protein, which may result in different outcomes.

Here, we demonstrate that different from the DNA without rearrangement and ubiquitination that elicited low-titer antibody and low-level T cell responses, or the full-length S protein that induced high-titer antibody but low-level T cell responses, the rearranged and ubiquitinated DNA vaccine targeting the original SARS-CoV-2 S protein induced robust specific T cell responses without antibody responses, which provided protection against SARS-CoV-2 (original strain), extending survival, and reducing weight loss in B6-backgrounded hACE2-Tg mice. It also cross-protected mice against the Omicron variant, with significantly reduced viral titers in the lungs of BALB/c mice. Notably, the adjuvants alone without DNA did not show protective efficacy against SARS-CoV-2 infection (data not shown).

We have also found that both SARS-CoV-2 Ub-S DNA vaccine–induced CD4^+^ and CD8^+^ T cells play a critical role in the protection against SARS-CoV-2 infection, as depletion of either one resulted in significant loss of vaccine-mediated protection. One important function of CD4^+^ T cells is to provide help for the generation of CD8^+^ CTLs, in particular for memory CTLs. We believe that CD4^+^ T cell effector functions may also be important for direct protection because the depletion of CD4^+^ T cells was done just before the viral challenge. Most likely, CD4^+^ T cells mediated their effects via cytokines such as IFN-γ, which may directly inhibit SARS-CoV-2 replication as shown by other research groups (44). CD8^+^ T cells may provide protection via cytolytic activity. CD4^+^ T cells seem to provide better protection than CD8^+^ T cells, most likely due to IFN-γ. As the vaccine did not induce any antibody responses, it should not fully prevent SARS-CoV-2 infection (antibodies may neutralize cell-free SARS-CoV-2 to effectively prevent infection), but prevent severe symptoms.

In this study, we used a ubiquitination strategy to promote degradation of SARS-CoV-2 S in the proteasome, as we did in a ZIKV DNA vaccine (23). Although the degradation of SARS-CoV-2 S protein is clearly shown in Figure 1, the degradation was not complete (the S protein band can still be seen without the proteasome inhibitor). This is different from the ZIKV DNA vaccine, because degradation of NS3 is almost complete. This may explain why the efficacy of the ZIKV vaccine is more dependent on CD8^+^ T cells rather on CD4^+^ T cells (23). We believe that the S protein that was not degraded in the proteasome and then leaked into the MHC class II processing pathway, resulting in generation of specific CD4^+^ T cells for protection.

Neutralizing activity against newer SARS-CoV-2 variants is reduced in the sera from individuals vaccinated with the SARS-CoV-2 vaccines targeting the original S protein (Wuhan clone) (45, 46). However, mRNA vaccine–induced T cells responded similarly to different SARS-CoV-2 variants, e.g., people vaccinated with mRNA vaccines targeting the original S are equally reactive to the Omicron strain (47). We performed the analyses on the mutations of MHC class I– and II–restricted epitopes of the S proteins and found that most of the epitopes are preserved. Furthermore, convalescent COVID-19 patients presented dominant CTL responses to S, membrane (M), and nucleocapsid (N) proteins (48–51); T cell memory responses to SARS-CoV-2 infection or vaccination have been shown to persist (52–54). These data support the idea that the current vaccines should provide protection against future variants. However, it is unclear whether T cell responses alone prevent breakthrough infections. Notably, mRNA-vaccinated individuals with induction of both T cells and neutralizing antibodies against SARS-CoV-2 can get infected. Mostly likely, insufficient amounts or lack of upper respiratory tract mucosal IgA and tissue-resident T cells against SARS-CoV-2 may lead to breakthrough infections. Nonetheless, the T cells should prevent severe symptoms, as suggested in the mouse models.

This study demonstrated the protective efficacy of the Ub-S DNA vaccine against the SARS-CoV-2 wild-type strain, mouse-adapted strain, and an Omicron variant in adult mice at young, mature, and middle age. Additional work will be warranted to evaluate its protective efficacy against future dominant variants in geriatric mice, by determining the role of vaccine-induced T cells in preventing SARS-CoV-2–caused severe diseases.

In summary, this study indicates that the current vaccines targeting the S protein may still provide protection against future variants of concern because most T cell epitopes presented by MHC class I and II are conserved. In addition, it also demonstrates that T cell responses alone without antibodies provide protection, a foundation for the development of a universal coronavirus vaccine, as conserved proteins such as M and N can be also used as the target antigens to induce T cell responses.

## Methods

### Construction of the vaccine and control plasmids.

The ubiquitinated and rearranged S gene construct (Ub-S DNA) and ubiquitinated original (unmodified) S gene construct (Ub-S Unmodified) for SARS-CoV-2 S DNAs were made as previously described, with some modifications (23). To construct the Ub-S DNA, the SARS-CoV-2 S protein sequence from the SARS-CoV-2 original strain (GenBank accession number QHR63250.2) was rearranged, and a ubiquitin sequence was added to the front of the rearranged S (see Results section). A Kozak sequence was placed ahead of the Ub/S open-reading frame to ensure efficient transcription of the plasmid. Upstream the Kozak sequence, linker DNA as well as an EcoRI restriction site were placed to facilitate proper cloning of the gene segment into the vector of interest. Downstream from the rearranged Ub/S gene sequence, a stop codon was placed along with linker DNA and a NotI restriction site. This nucleotide sequence was ordered from GenScript. The S gene was delivered in a vector in lyophilized form. Plasmid DNA was resuspended in Molecular Biology Grade water (Thermo Fisher Scientific) to a concentration of approximately 200 ng/μL. DNA was digested with FastDigest (FD) EcoRI and NotI for 15 minutes in a 37°C heat block. The digested DNA was then loaded directly onto a 1% agarose gel and allowed to run for 40 minutes at 80 V. The Ub-S DNA with correct size (4,170 bp) was exposed to 440 nm UV light in the imaging room and excised. The gel slice was placed into a clear, 1.5 mL microcentrifuge tube and the purified DNA was extracted using a QIAquick Gel Extraction Kit (Qiagen). This gene fragment was stored in a –20°C freezer. The pVAX1 vector (Thermo Fisher Scientific) was digested with FD EcoRI and FD NotI, run in a gel, and extracted using the same kit. The Ub-S gene fragment was ligated using a 3:1 ratio of insert DNA (Ub-S) to vector DNA (pVAX1) at room temperature in the presence of T4 DNA ligase. To construct the Ub-S unmodified DNA, the ubiquitin sequence was added to the front of the original S sequence and cloned into pVAX1 as above. To construct the S gene without ubiquitin, a sequence encoding an N-terminal CD5 signal peptide was added to the front of the original S sequence and cloned into pVAX1 as above.

### Bacterial transformation.

Previously made chemically competent DH5α *E*. *coli* were removed from –80°C storage and thawed on ice for 25 minutes. After adding the plasmids, the *E*. *coli* were incubated on ice for another 30 minutes. Heat shock transformation was conducted by placing the tube into a 42°C heat block for 45 seconds and back on ice for 2 minutes. One milliliter of SOC media was added to the *E*. *coli* and the bacteria allowed to recover in a shaking incubator at 37°C for 45 minutes. The *E*. *coli* were then spun down at 7,800*g* for 2 minutes, resuspended in 200 μL SOC media, and streaked on an LB kanamycin (Kan^+^) plate. Once dry, the plate was inverted and incubated overnight at 37°C. Colonies from the plate were inoculated into 2 mL LB Kan^+^ media, and DNA purified by miniprep using a QIAprep Spin Miniprep Kit according to the manufacturer’s instructions (Qiagen). The plasmid was subsequently redigested with FD EcoRI and FD NotI. Upon observance of correct band digestion, the plasmid was sent out for sequencing (ACGT, Inc) for confirmation of the correct insertion.

### Plasmid transfection.

293T cells (ATCC) were cultured in Dulbecco’s modified Eagle medium (DMEM) with 10% fetal bovine serum (FBS) and 1% penicillin/streptomycin. Cells were split upon reaching 90% confluence (every 2–3 days). Upon sufficient generation of 293T cells, 10 μg of the plasmid was transfected using PEI transfection reagent overnight. Media were removed the next morning and cells were cultured in DMEM. For subsequent experiments using proteasome inhibitor, MG132 (Sigma-Aldrich) was added to cell culture medium at 50 μM overnight at 12, 36, or 60 hours after transfection.

### Western blot.

293T cells were cultured, transfected, and treated with proteasome inhibitor as described above. After overnight treatment with 50 μM MG132, 293T cells were treated with 0.25% trypsin for 5 minutes at 37°C in 5% CO_2_. 293T cells were resuspended in DMEM, centrifuged at 1,200 rpm for 5 minutes, and cell pellets were resuspended in RIPA buffer with freshly added proteinase inhibitor. Total protein concentration was calculated via Bradford Assay (Bio-Rad), and 6× SDS Loading Buffer was added to 20 μg of total protein. Protein was denatured at 100°C for 10 minutes and then placed on ice for 2–3 minutes. Proteins were run in a 12% polyacrylamide gel for 20 minutes at 80 V and for 40 minutes at 120 V, or until the dye front reached the bottom of the gel. Proteins from the polyacrylamide gel were transferred to a nitrocellulose membrane using an iBlot Gel Transfer Device (Thermo Fisher Scientific). The membranes was blocked in 5% blocking buffer (5% nonfat milk in PBS) for 1 hour, washed in PBS–Tween 20 (PBS-T), and blocked in 5% blocking buffer containing a 1:1,000 dilution of anti–SARS-CoV-2 S antisera overnight (32). The next day, the membrane was washed with PBS-T for 20 minutes followed by blocking in 5% blocking buffer with secondary horseradish peroxidase–conjugated (HRP-conjugated) goat anti-rabbit antibody (BioLegend) for 1 hour. The membrane was washed in PBS-T for 20 minutes and then exposed to 10 mL chemiluminescent substrate for 3 minutes, in the absence of light. Proteins were visualized using a FluorChem E system (ProteinSimple).

### Preparation of recombinant proteins.

SARS-CoV-2 full-length S protein and its fragments were prepared as previously described (32, 55). Briefly, DNA sequences of SARS-CoV-2 S or its fragments (RBD, NTD, or S2) were amplified by PCR using a plasmid encoding codon-optimized S protein of the SARS-CoV-2 original strain (GenBank accession number QHR63250.2). The recombinant plasmids were constructed by inserting respective PCR fragments (S containing a C-terminal foldon trimeric sequence and His_6_ tag; RBD, NTD, or S2 containing a C-terminal His_6_ tag with or without a foldon tag) into a pLenti expression vector. This was followed by transfecting each recombinant plasmid into 293T cells and purifying each protein from cell culture supernatants using Ni-NTA Superflow (Qiagen).

### Immunization of mice with vaccines and sample collection.

SARS-CoV-2 vaccines were used to immunize mice according to the following 3 immunization protocols (23, 32). First, female BALB/c mice (6–8 weeks old) were intramuscularly (i.m.) vaccinated with Ub-S DNA (10 μg/mouse) plus imiquimod adjuvant (20 μg/mouse; InvivoGen). Mice immunized (i.m.) with a mammalian cell–expressed SARS-CoV-2 full-length S protein (10 μg/mouse) plus Alhydrogel Alum (500 μg/mouse) and MPL 10 μg/mouse) adjuvants (InvivoGen) were used as a vaccine control, and mice injected with PBS were included as a background control. The immunized mice were boosted twice with the same immunogens and adjuvants at 3-week intervals. Ten days after the third immunization, sera and splenocytes were collected for detection of SARS-CoV-2 S–, NTD–, RBD–, S1–, or S2–specific IgG antibodies and S–specific T cell responses, respectively. Second, female B6 mice (6–8 weeks old) were immunized (i.m.) with Ub-S DNA, original S DNA control, full-length S protein control (10 μg/mouse), or PBS control in the presence of imiquimod adjuvant and Alum plus MPL adjuvants, respectively, and boosted with the same immunogens and adjuvants twice at 3 weeks and once at 6 months. Sera were collected 10 days after the third immunization as above to test for SARS-CoV-2 S–, NTD–, RBD–, S1–, or S2–specific IgG antibodies, and splenocytes were collected 10 days after last immunization to detect S–specific T cell responses. Third, female BALB/c mice (10 weeks old) were immunized with imiquimod-adjuvanted Ub-S DNA, original S DNA control, full-length S protein control (10 μg/mouse), or PBS background control, and boosted twice at 3-week intervals. Sera collected before SARS-CoV-2 challenge were tested for IgG antibodies specific for SARS-CoV-2 S and its fragments (NTD, RBD, S1, or S2) as described below.

### ELISA.

SARS-CoV-2–specific IgG antibodies were detected by ELISA using the harvested mouse sera (32, 55–57). Briefly, ELISA plates were precoated with the purified SARS-CoV-2 NTD, RBD, S2, or full-length S, as well as S1 (BEI Resources), protein (1 μg/mL) at 4°C overnight, followed by blocking with PBS-T containing 2% fat-free milk at 37°C for 2 hours. The plates were washed 3 times with PBS-T, and then sequentially incubated with serially diluted mouse sera and HRP-conjugated goat anti–mouse IgG antibody (Fab-specific: 1:5,000; Sigma-Aldrich) at 37°C for 1 hour. The plates were further incubated with the substrate 3,3′,5,5′-tetramethylbenzidine (TMB) (Sigma-Aldrich), and then H_2_SO_4_ (1N) to stop the reaction. Absorbance at 450 nm (A450) was measured using a Cytation 7 Microplate Multi-Mode Reader (BioTek Instruments).

### Flow cytometry.

Flow cytometry analysis was performed to detect SARS-CoV-2 S–specific CD4^+^ and CD8^+^ T cell responses in the above-collected mouse splenocytes (58). Briefly, splenocytes (1 × 10^6^ cells/well) were incubated with a mixture of peptides predicted to be mouse CTL and T helper cell epitopes in the SARS-CoV-2 S protein (final concentration 5 μg/mL/peptide; Supplemental Tables 3 and 4), and cultured at 37°C. Forty-two hours later, the cells were restimulated with the same peptides in the presence of mouse IL-2 (1 μg; R&D Systems) and Brefeldin A (5 μg/mL; Sigma-Aldrich). Six hours later, the cells were washed with PBS and stained for surface markers using anti–mouse CD45–Alexa Fluor 700 (147716, BioLegend), anti–mouse CD8-PerCP/Cy5.5 (100734, BioLegend), and anti–mouse CD4-FITC (553729, BD Pharmingen) antibodies. After fixation and permeabilization, the cells were further stained for intracellular markers using anti–mouse IFN-γ–PE (554412, BD Pharmingen) and anti–mouse-TNF-α–BV421 (506328, BioLegend) antibodies, followed by analysis using a CytoFLEX flow cytometer (Beckman Coulter Life Sciences).

### Evaluation of vaccine efficacy in the immunized mice.

The hACE2-Tg mice (6- to 8-week-old females or 4- to 6-month-old males and females), BALB/c mice (10-week-old females), and C57BL/6 (B6) mice (8- to 9-month-old males and females) were respectively immunized with SARS-CoV-2 Ub-S DNA, original S DNA, full-length S protein, and/or PBS as described above. Four separate experiments were performed to evaluate the efficacy of SARS-CoV-2 vaccines in the immunized mice, as described previously (23, 32, 55). First, 2 weeks after the last dose, hACE2-Tg mice respectively immunized with Ub-S DNA (imiquimod adjuvant), full-length S protein control (Alum + MPL adjuvants), or PBS, were intranasally (i.n.) challenged with SARS-CoV-2 (human strain 2019n-CoV/USA-WA1/2020, 5,000 PFU/mouse, 50 μL/mouse), and observed for survival and weight changes daily for 14 days. Second, 2 weeks after the last dose, B6 mice respectively immunized with Ub-S DNA, full-length S protein control, or PBS, as described above, were challenged (i.n.) with SARS2-CoV-2 (mouse-adapted N501YMA_30_, 5,000 PFU/mouse, 50 μL/mouse). The challenged mice were sacrificed 2 days p.i., and lung tissues were detected for viral titers as described below. Third, 4 weeks after the last dose, BALB/c mice that were respectively immunized with imiquimod-adjuvanted Ub-S DNA, original S DNA control, or full-length S protein control, as well as PBS background control (as described above), were challenged (i.n.) with SARS-CoV-2 Omicron BA.5 (50,000 PFU/mouse, 50 μL/mouse), and evaluated for viral titers in the lungs 2 days after challenge. Fourth, 8 weeks after the last dose of imiquimod-adjuvanted Ub-S DNA vaccine and PBS, immunized hACE2-Tg mice were intraperitoneally injected with anti–mouse CD4 (IgG2b, for depleting CD4^+^ T cells; BP0003-1, Bio X Cell), anti–mouse CD8a (IgG2b, for depleting CD8^+^ T cells; BP0061, Bio X Cell) (200 μg/mouse), or IgG2b isotype control (without depleting CD4^+^ and CD8^+^ T cells; BP0090, Bio X Cell) antibody, respectively, on days –2, –1, and 1 after SARS-CoV-2 challenge (23), and the mice were challenged (i.n.) with SARS-CoV-2 (2019n-CoV/USA-WA1/2020, 2,000 PFU/mouse, 50 μL/mouse). The challenged mice were observed for survival and weight changes daily for 14 days. The challenged mice with greater than 30% weight loss and significant clinical symptoms were humanely euthanized.

### SARS-CoV-2 plaque assay.

SARS-CoV-2 titers in the challenged mouse lung were detected by plaque assay (55, 56). Specifically, supernatants from the homogenized lung tissues were serially diluted in DMEM and incubated with Vero E6 cells (ATCC) preplated in 12-well plates at 37°C for 1 hour in 5% CO_2_ with gentle rocking every 15 minutes. After removing the inoculum, the plates were overlaid with 1.2% agarose containing 4% FBS. After further incubation for 2 days, overlays were removed, and plaques were visualized by staining with 0.1% crystal violet.

### Statistics.

Statistical significance among different groups was assessed by ordinary 1-way ANOVA with Tukey’s (Figures 2 and 3) or Dunnett’s (Figures 4–6) multiple-comparison test using GraphPad Prism 9 software. A *P* value of less than 0.05 was considered significant: *P < 0.05, **P < 0.01, ***P < 0.001.

### Study approval.

Male and female BALB/c, B6, and hACE2-Tg mice (Jackson Laboratory or self-breeding) were used in this study. The animal protocols were approved by Institutional Animal Care and Use Committees (IACUCs) of the New York Blood Center (New York, New York), Georgia State University (Atlanta, Georgia), and the University of Iowa (Iowa City, Iowa). The mouse-related experiments were carried out according to the guidelines of the approved IACUC protocols.

### Data and materials availability.

All data are available in the main text, the supplementary materials, or Supporting Data Values file.

## Author contributions

LQ and LD designed the project and vaccines and wrote and revised the manuscript, with input from all authors. RC, JD, and GW designed and constructed the vaccines and controls. RC and NJ tested protein expression. JS, XZ, WT, and XG prepared the vaccines and figures, immunized animals, tested vaccine immunogenicity, and analyzed the data. JZ, AKV, and AEO performed viral challenge experiments. YW and FL prepared the proteins and analyzed the data. RC analyzed the T cell epitope mutations. LQ, LD, and SP evaluated the data and supervised the project.

## Supplementary Material

Supplemental data

Unedited blot and gel images

Supporting data values

## Version 1 01/23/2024

In-Press Preview

## Version 2 03/08/2024

Electronic publication
