# Supplementary material for: A T cell–based SARS-CoV-2 spike protein vaccine provides protection without antibodies
Source: JCI Insight. 2024 Jan 23;9(5):e155789. doi: 10.1172/jci.insight.155789 (PMC10972590; doi:10.1172/jci.insight.155789)
Supplement: Supplemental data [file jciinsight-9-155789-s110.pdf]

Supplementary Materials for

A T cell-based SARS-CoV-2 spike protein vaccine provides protection without antibodies

Juan Shi[1#], Jian Zheng[2,7#], Xiujuan Zhang[3#], Wanbo Tai[3#], Ryan Compas[4#], Jack Deno[4], Natalie Jachym[4], Abhishek K Verma[2], Gang Wang[1], Xiaoqing Guan[1], Abby E Odle[2], Yushun Wan[5], Fang Li[5,6], Stanley Perlman[2*], Liang Qiao[4*] and Lanying Du[1*]

[1]Institute for Biomedical Sciences, Georgia State University, Atlanta, GA, USA;
[2]Department of Microbiology and Immunology, and Department of Pediatrics, University of Iowa, Iowa City, IA, USA;
[3]Lindsley F. Kimball Research Institute, New York Blood Center, New York, NY, USA;
[4]Department of Microbiology and Immunology, Stritch School of Medicine, Loyola University Chicago, Maywood, IL, USA;
[5]Department of Pharmacology, University of Minnesota Medical School, Minneapolis, MN, USA;
[6]Center for Coronavirus Research, University of Minnesota, Minneapolis, MN, USA;
[7]Present address: Department of Microbiology and Immunology, Center for Predictive Medicine, University of Louisville, Louisville, KY, USA.

[#]These authors contributed equally.
*Corresponding senior authors.
Mailing addresses:
Stanley Perlman, Departments of Microbiology and Immunology, and Pediatrics, University of Iowa, Iowa City, IA 52242, USA.
Phone number: 319-335-8549.
Email: stanley-perlman@uiowa.edu.
Liang Qiao, Department of Microbiology and Immunology, Stritch School of Medicine, Loyola University Chicago, 2160 South First Avenue, Maywood, IL 60153, USA.
Phone number: 708-327-3481.
Email: lqiao@luc.edu.
Lanying Du, Institute for Biomedical Sciences, Georgia State University, Atlanta, GA 30303, USA.
Phone number: 404-413-3578.
Email: ldu3@gsu.edu.

Supplementary Figures

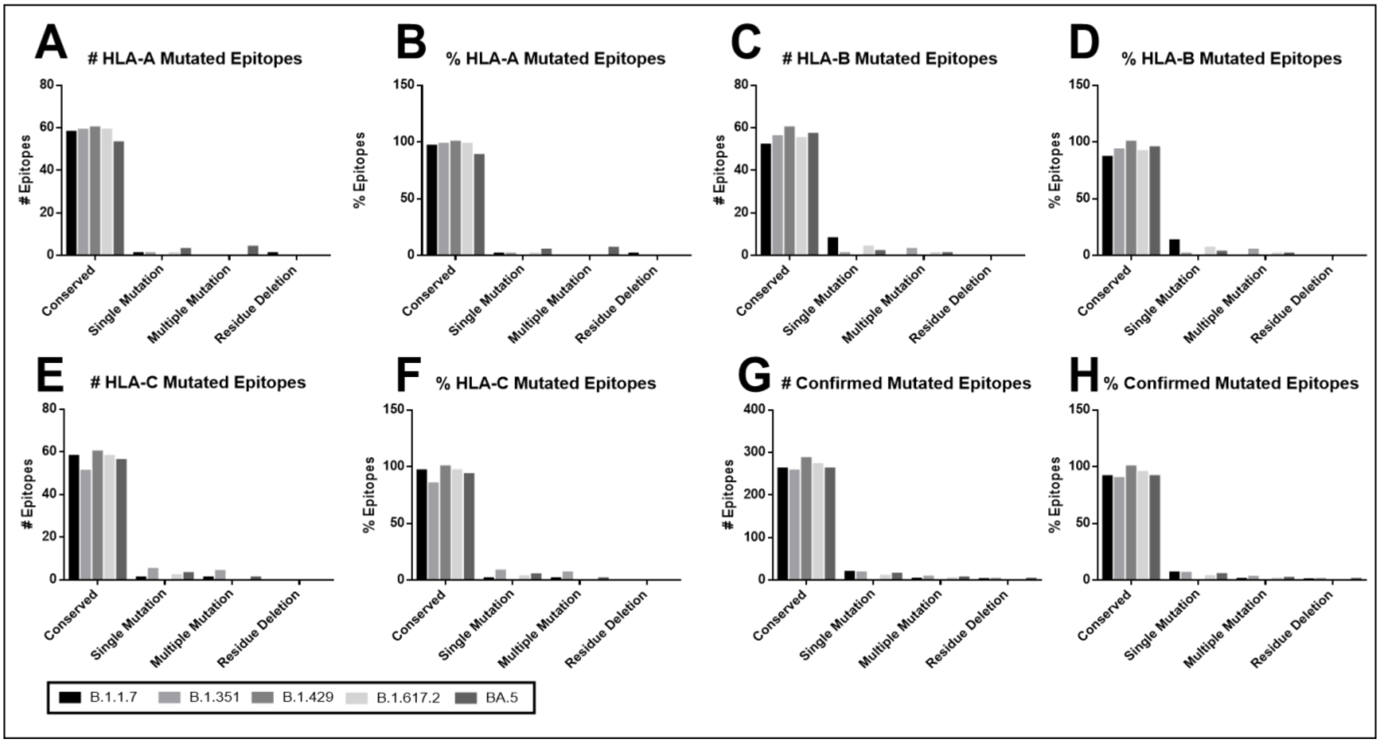

Figure S1. MHC class I epitope mutations in variants of concern. The S proteins of five variant strains were aligned with SARS-CoV-2 S protein of original strain to search for the predicted and confirmed CD8 T-cell epitope mutations. The results of the top six alleles with the highest occurring frequencies (see **Table S1**) for HLA-A, HLA-B, and HLA-C are shown above (**A-F**). Ten epitopes with the highest scoring IC_{50} values for each allele were chosen using TepiTool, a prediction analysis resource from the Immune Epitope Database (IEDB). Total 60 epitopes from HLA-A, B and C, respectively, were used for analysis for mutations. Total 286 confirmed epitopes from IEDB were analyzed for mutations for all MHC class I alleles (**G, H**). Mutations found were sorted by single residue mutations, single residue deletions, or multiple mutations that encompass any combination of single residue mutations or deletions.

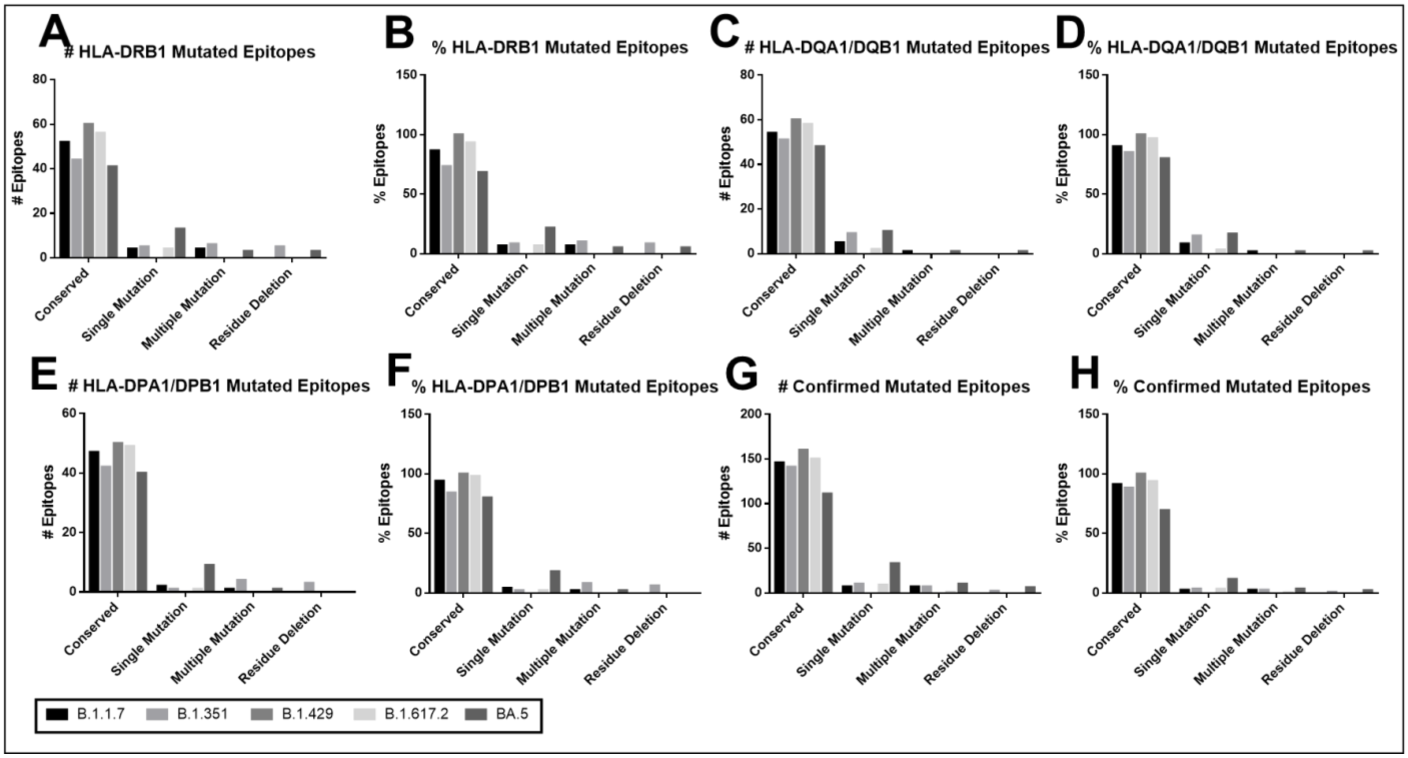

Figure S2. MHC class II epitope mutations in variants of concern. S proteins of five variant strains were aligned with SARS-CoV-2 S protein to search for predicted and confirmed $CD4^+$ T-cell epitope mutations. The results of the top six alleles with the highest occurring frequencies (see **Table S2**) for HLA-DRB1, HLA-DQA1/DQB1, and HLA-DPA1/DPB1 are shown above **(A-F).** Ten epitopes of S protein from the original strain with the highest scoring IC_{50} values for each allele were chosen using TepiTool, a prediction analysis resource from the Immune Epitope Database (IEDB). Total 60 epitopes from HLA-DR, DQ and DP, respectively, were used for analysis for mutations. Total 160 confirmed epitopes from IEDB were analyzed for mutations for all MHC class II alleles (**G, H**). Mutations found were sorted by single residue mutations, single residue deletions, or multiple mutations that can encompass any combination of single residue mutations or deletions.

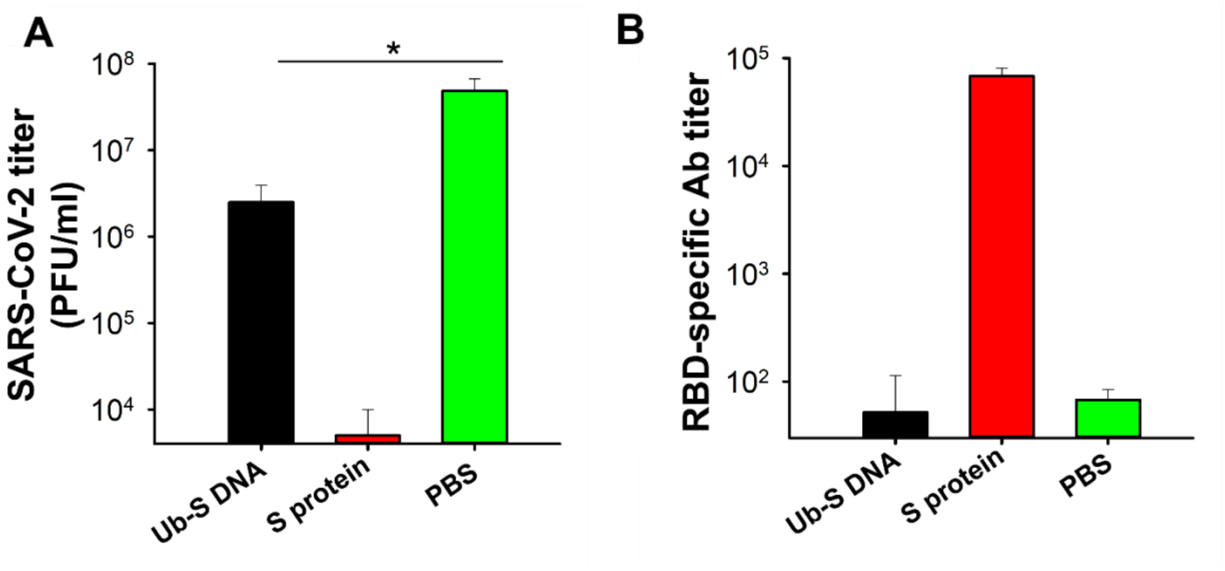

Figure S3. SARS-CoV-2 DNA vaccine induced protection against SARS-CoV-2 challenge with reduced viral titers. B6 mice were immunized with Ub-S DNA vaccine, full-length S protein vaccine control, or PBS control. Mouse sera were collected at two weeks after last dose of immunization, and the mice were I.N. infected with SARS-CoV-2 (mouse-adapted strain $N501YMA_{30}$, 5,000 PFU/mouse). (A) SARS-CoV-2 titers were detected in the lung by plaque assay after 2 days p.i. Statistical difference between SARS-CoV-2 Ub-S DNA and PBS control was performed using unpaired student *t* test and GraphPad Prism 9 statistical software. * indicates $P < 0.05$. (B) SARS-CoV-2 RBD-specific IgG antibodies were detected in the sera of mice before SARS-CoV-2 infection. The data are presented as mean ± s.e.m. of mice in each group (n = 4). Experiments were repeated once, and similar results were obtained.

Supplementary Tables

Table S1

HLA-A, HLA-B and HLA-C allele (AF) and cumulative frequencies (cF) in the sample of 111 unrelated blood donor volunteers from East Croatia.

	Allele HLA-A	n	AF	cF	Allele HLA-B	n	AF	cF	Allele HLA-C	n	AF	cF
1	A*02:01:01	72	32.43%	32.43%	B*51:01:01	19	8.56%	8.56%	C*04:01:01:01	31	13.96%	13.96%
2	A*01:01:01:01	27	12.16%	44.59%	B*08:01:01	18	8.11%	16.67%	C*07:01:01	25	11.26%	25.23%
3	A*03:01:01:01	26	11.71%	56.31%	B*35:01:01	18	8.11%	24.77%	C*02:02:02	21	9.46%	34.68%
4	A*24:02:01:01	26	11.71%	68.02%	B*07:02:01	15	6.76%	31.53%	C*07:02:01	18	8.11%	42.79%
5	A*11:01:01:01	22	9.91%	77.93%	B*35:03:01	15	6.76%	38.29%	C*12:03:01	16	7.21%	50.00%
6	A*32:01:01:01	8	3.60%	81.53%	B*18:01:01	12	5.41%	43.69%	C*06:02:01:01	15	6.76%	56.76%

Sourced from:
https://www.nature.com/articles/s41598-020-62175-9/tables/1

Table S2

HLA-DRB1, HLA-DQA1 and HLA-DQB1 allele (AF) and cumulative frequencies (cF) in the sample of 111 unrelated blood donor volunteers from East Croatia.

	Alelle HLA-DRB1	n	AF	cF	Allele HLA-DQA1	n	AF	cF	Allele HLA-DQB1	n	AF	cF
1	DRB1*16:01:01	30	13.51%	13.51%	DQA1*01:02:02	35	15.77%	15.77%	DQB1*05:02:01:01	35	15.77%	15.77%
2	DRB1*01:01:01	24	10.81%	24.32%	DQA1*03:01:01	16	7.21%	22.97%	DQB1*03:01:01:03	33	14.86%	30.63%
3	DRB1*07:01:01	22	9.91%	34.23%	DQA1*02:01:01:01	15	6.76%	29.73%	DQB1*05:01:01:03	23	10.36%	40.99%
4	DRB1*15:01:01	15	6.76%	40.99%	DQA1*01:03:01:02	13	5.86%	35.59%	DQB1*02:01:01	17	7.66%	48.65%
5	DRB1*03:01:01:01	12	5.41%	46.40%	DQA1*05:01:01:02	12	5.41%	40.99%	DQB1*03:02:01:01	16	7.21%	55.86%
6	DRB1*11:01:01:01	10	4.50%	50.90%	DQA1*01:02:01:01	10	4.50%	45.50%	DQB1*02:02:01:01	15	6.76%	62.61%

Sourced from:
https://www.nature.com/articles/s41598-020-62175-9/tables/2

Table S3

Peptides covering the SARS-CoV-2 S protein for stimulation of splenocytes in BALB/c mice

Peptide name	Sequence
2019CoV-19	TVLPPLTDEMIAQYTSALL
2019CoV-20	LQIPFAMQMAYRFNGIGVTQ
2019CoV-21	NVLYENQKLIANQFNSAIGK
2019CoV-22	IQDSLSSTASALGKLQDVVN
2019CoV-23	LQDVVNQNAQALNTLVKQLS
2019CoV-24	KQLSSNFGAISSVLNDILSR
2019CoV-25	MSFPQSAPHGVVFLHVTYVP
2019CoV-26	CGPKKSTNLVKNKCVNFNFN
2019CoV-27	VNFNFNGLTGTGVLTESNKK
2019CoV-28	NSPRRARSVASQSIIAYTMSL
2019CoV-29	SIIAYTMSLGAENSVAYSNNSI
2019CoV-30	LPPAYTNSFTRGVYYPDKVF
2019CoV-31	STQDLFLPFFSNVTWFHAIHVS
2019CoV-32	SGTNGTKRFDNPVLPFNDGVYF
2019CoV-33	YSSANNCTFEYVSQPFLMDL
2019CoV-34	WTAGAAAYYVGYLQPRTFLL
2019CoV-35	IYQTSNFRVQPTESIVRFPN

Table S4

Peptides covering the SARS-CoV-2 S protein for stimulation of splenocytes in B6 mice

Peptide name	Sequence
7 of 181	FRSSVLHSTQDLFLPFF
8 of 181	STQDLFLPFFSNVTWFH
11 of 181	SGTNGTKRFDNPVLPFN
15 of 181	NIIRGWIFGTTLDSKTQ
31 of 181	NLVRDLPQGFSALEPLV
32 of 181	QGFSALEPLVDLPIGIN
33 of 181	PLVDLPIGINITRFQTL
38 of 181	AGAAAYYVGYLQPRTFL
57 of 181	TNVYADSFVIRGDEVRQ
67 of 181	PFERDISTEIYQAGSTP
68 of 181	TEIYQAGSTPCNGVEGF
69 of 181	STPCNGVEGFNCYFPLQ
73 of 181	YQPYRVVVLSFELLHAP
74 of 181	VLSFELLHAPATVCGPK
77 of 181	LVKNKCVNFNFNGLTGT
94 of 181	GAEHVNNSYECDIPIGA
99 of 181	VASQSIIAYTMSLGAEN
117 of 181	SKRSFIEDLLFNKVTLA
118 of 181	DLLFNKVTLADAGFIKQ
150 of 181	GKGYHLMSFPQSAPHGV
153 of 181	VTYVPAQEKNFTTAPAI
154 of 181	EKNFTTAPAICHDGKAH
172 of 181	IDLQELGKYEQYIKWPW
173 of 181	KYEQYIKWPWYIWLGFI
