# Supplementary figures and images for: A T cell–based SARS-CoV-2 spike protein vaccine provides protection without antibodies
Source: JCI Insight. 2024 Jan 23;9(5):e155789. doi: 10.1172/jci.insight.155789 (PMC10972590; doi:10.1172/jci.insight.155789)

### Full Unedited Blots for Fig. 1C

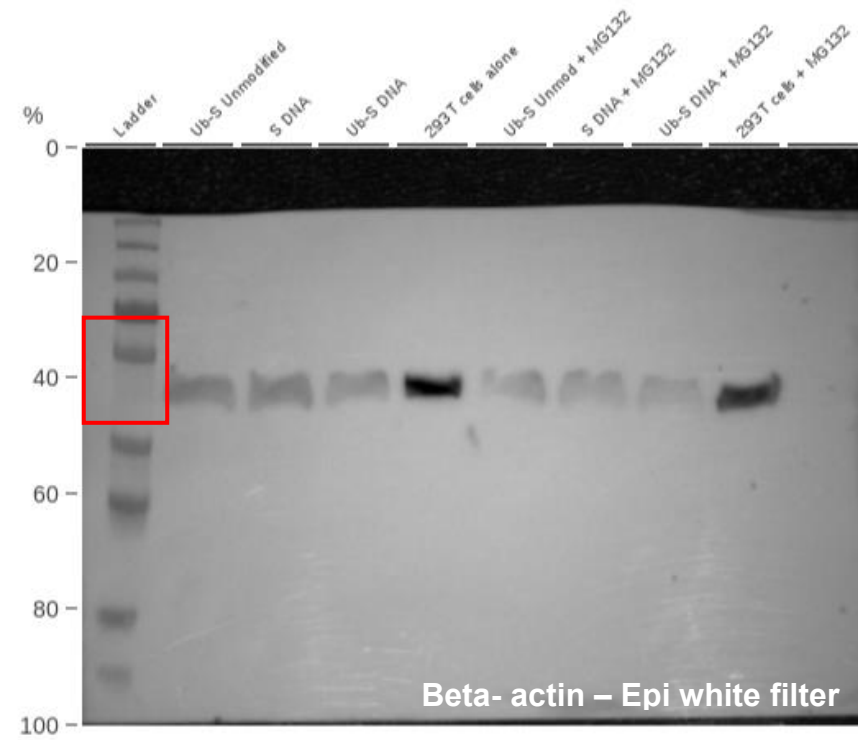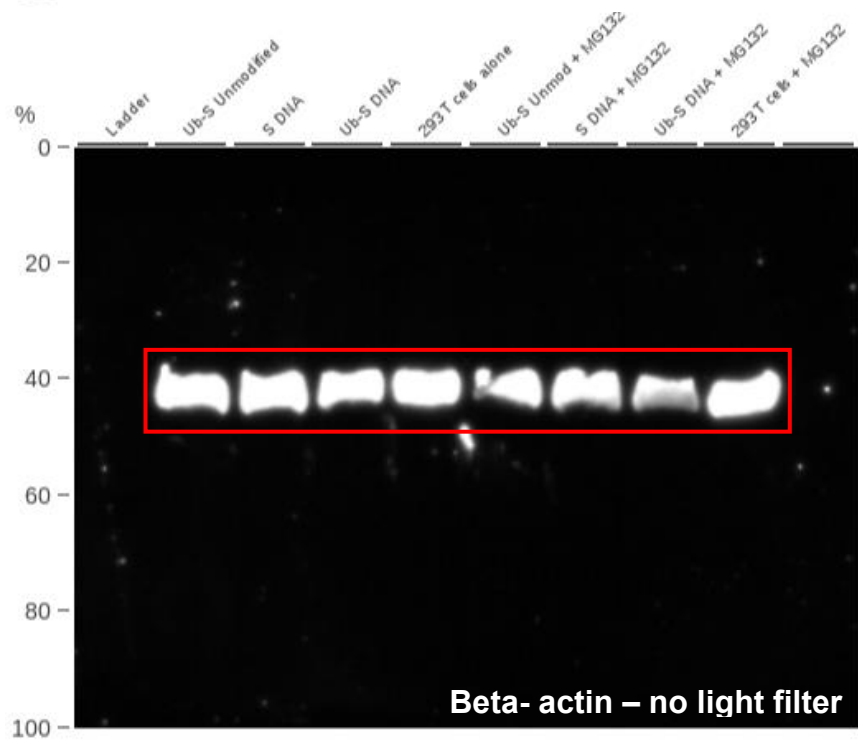

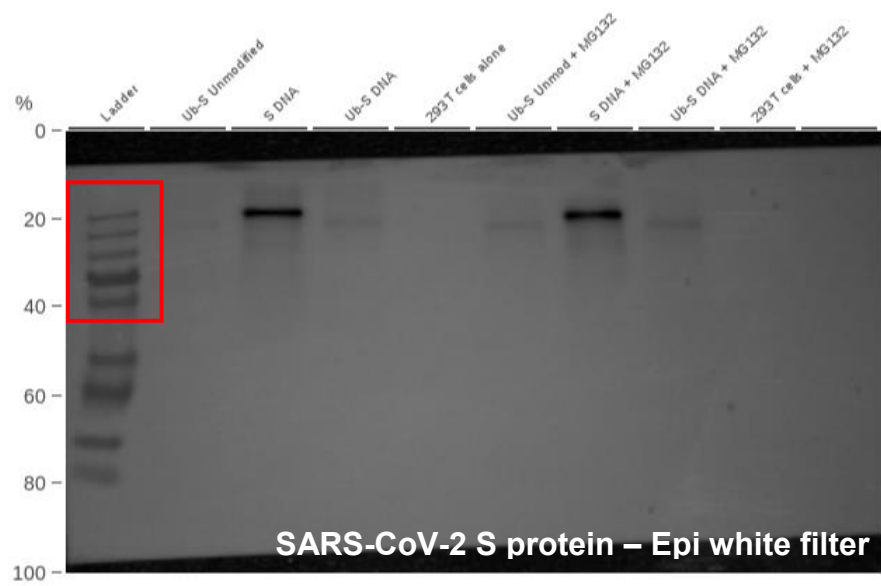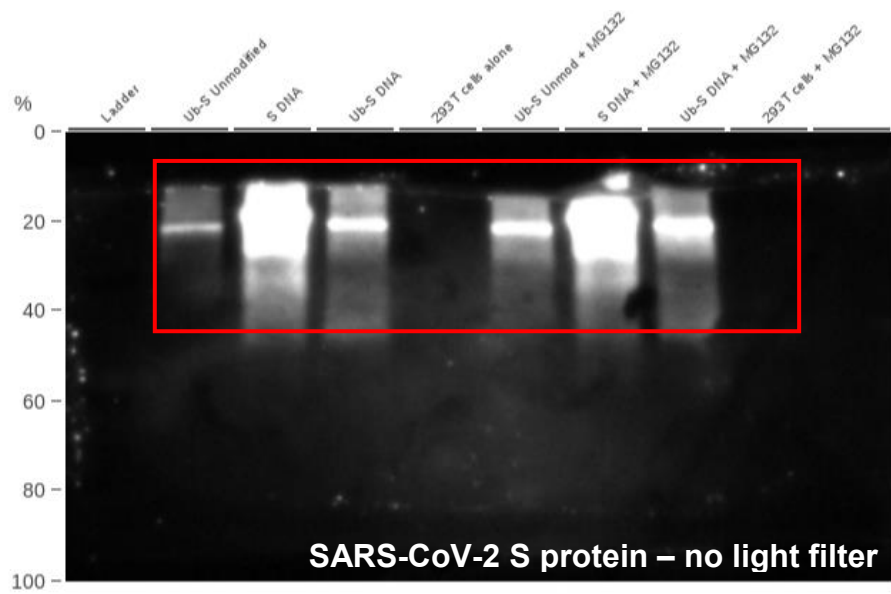

Supplement: Unedited blot and gel images [file jciinsight-9-155789-s111.pdf]
